# Socio-Economic Marginalization and Compliance Motivation Among Students and Freeters in Japan

**DOI:** 10.3389/fpsyg.2019.00312

**Published:** 2019-02-26

**Authors:** I-Ting Huai-Ching Liu, Yukiko Uchida, Vinai Norasakkunkit

**Affiliations:** ^1^Graduate School of Human and Environmental Sciences, Kyoto University, Kyoto, Japan; ^2^Kokoro Research Center, Kyoto University, Kyoto, Japan; ^3^Department of Psychology, Gonzaga University, Spokane, WA, United States

**Keywords:** Hikikomori, NEET, freeter, marginalization, self-consistency, social proof, compliance motivation

## Abstract

This study examines the compliance motivation of students and Freeters when facing a marginalization risk situation evoked by priming. Freeter (part-time employers), NEET (not in education, employment, or training), and Hikikomori (social withdrawal) represent the socio-economically marginalized population in Japan. People at higher risk of becoming NEET and Hikikomori have shown a motivation pattern deviant from mainstream Japanese culture, including lower willingness to conform to in-group members, thus showing less cultural fit (Norasakkunkit and Uchida, 2014). In this study we explore the effect of the macro socio-economic situation (job-hunting prospects being good or bad) on individual's compliance motivation in both students and Freeters. Sixty-five Kyoto University students and 74 Freeters were randomly assigned to one of the two priming conditions (marginalization risk or non-marginalization) before completing the NEET-Hikikomori Risk (NHR) scale and measurements of compliance motivation to conform to in-group members or to be self-consistent (Cialdini et al., 1999). Twenty-three control group students and 22 control group Freeters were also recruited online for comparison. Results showed that marginalization risk priming led to lower tendency to be self-consistent among students, but did not lead to lower tendency to conform to in-group members. For Freeters, marginalization risk priming led to higher compliance motivation to conform to in-group members. The results confirmed the framework proposed by Toivonen et al. (2011) that both Freeters and students in Japan have ritualist reactions, continuing to maintain the cultural norms despite the difficulty of attaining the cultural goals.

## Introduction

In all societies, there are those who fit in the mainstream and those marginalized in the periphery. In Berry and Sam (1997) acculturation model, those who do not wish to maintain their own cultural identity and do not seek to engage with the dominant society have a marginalized orientation, lacking “cultural fit”. Applying the model to the Japanese context, the mainstream society consists of interdependent full-time workers who value group harmony and seniority (Markus and Kitayama, 1991; Kitayama et al., 2016). On the other hand, those with a marginalized orientation in the periphery are those who reject the mainstream Japanese cultural values but do not possess a different cultural identity, such as Freeter, NEET, and Hikikomori (Norasakkunkit and Uchida, 2011). The word “Freeter” in Japanese describes those who engage in part-time jobs only and do not seek a full-time, lifelong employment. “NEET” was first coined by Bynner and Parsons (2002) to describe those who are “not in education, employment, or training” in the UK. In the extreme case, “Hikikomori” (social withdrawal), a term first used in the academic field in 1986, describes people who avoid social interactions even with their family members, shutting themselves in their room for months or years (Kitao, 1986). The occurrence of Freeter, NEET, and Hikikomori has been increasing, and one explanation proposed was the economic recession and globalization in Japan, paired with the institutional reluctance to reform at the cost of decreasing young adults' motivation to participate in mainstream society (Toivonen et al., 2011). Following the explanation, this paper aims to elucidate the motivational responses of students and Freeters under different job-hunting prospects evoked by priming.

### Freeter, NEET, and Hikikomori in Japan

The prevalence of Freeter, NEET, and Hikikomori has risen in Japan over the past few decades. The number of articles related to Hikikomori in two major Japanese newspapers, Asahi Shinbun and Yomiuri Shinbun, has grown from merely 4 in 1985 to 794 in 2005 (Ishikawa, 2007). Likewise, the number of Freeters aged 20–24 has doubled from 1992 to 2002, and the number of NEETs has increased from 2.8 to 4.4% for males of the same age group (in Japan NEET does not include housewives and people who are seeking employment; Inui et al., 2006). A more recent survey has shown that 34.4% of the total Japanese population are in part-time employment, and that among the 15–34 years-olds, 6.4% are Freeters and 2.8% are NEETs (Norasakkunkit et al., 2012). Furlong (2008) argued that this growth is related to changes in the Japanese labor market since the economic recession in the 1990s, characterized by an expansion of non-regular employment opportunities and a contraction of regular employment opportunities. Because chances are smaller to enter the core labor market via the traditional system (a one-time job-hunting process before graduation from universities), in order to avoid distress young people tend to participate in the peripheral labor market, such as taking part-time jobs. Although there is a difference in the extent of non-conforming behavior, Freeter, NEET, and Hikikomori may have some overlap in their marginalized status in the society as a “marginalization spectrum” syndrome (Uchida and Norasakkunkit, 2015).

Norasakkunkit et al. (2012) claimed that the traditional Japanese societal structure and cultural practices which resist to globalizing economic pressures (such as price competition or labor market competition) serve to protect the senior elites at the cost of ostracizing the the younger generation. Due to globalization after millennium, there is pressure for societies to highlight individualism, meritocracy, competitiveness, high flexibility, and high mobility (Chiu et al., 2011), but Japanese institutional resistance to changing the traditional societal structure (sustaining permanent employment system, inflexible job-hunting process and seniority-based promotion) increases the rift between Japanese work cultures and globalizing trends. Those who succeed despite the diminishing chances of landing a permanent employment still partake in the mainstream interdependent society, but those who fail can be discouraged and demotivated. In the worst situation, this may lead to complete social withdrawal, as in the case of Hikikomori.

### Culturally Marginalized Behaviors

Uchida and Norasakkunkit (2015) examined the commonalities between NEET and Hikikomori, namely their values and attitudes that deviate from the dominant cultural practices, and developed a NEET-Hikikomori Risk Spectrum scale (NHR) to measure the risk of marginalization in a society. As sociological interviews have shown that NEET-Hikikomori risk was associated with culturally deviant motivation patterns (Saito, 2005), Norasakkunkit and Uchida (2011) showed that the tendency of becoming NEET and Hikikomori was negatively associated with persistence after failure feedback [mainstream Japanese behavior as in Heine et al. (2001)'s study], but was not significantly positively associated with persistence after success feedback. It also showed that students at higher risk of becoming NEET/Hikikomori scored lower in Singelis (1994)'s interdependent self-construal, but not higher in the independent self-construal.

Likewise, adapting the compliance motivation by Cialdini et al. (1999), Norasakkunkit and Uchida (2014) showed that high-risk students' willingness to comply (WTC) to a request when their in-group members complied to the same request (i.e., social proof scenario) was lower relative to low-risk students, which represents a deviation of high-risk students from the interdependent cultural norm. However, they did not have higher motivation to comply to maintain self-consistency either (i.e., dominant motivation pattern in independent cultures). Furthermore, the difference between high-risk and low-risk students' compliance in the social proof scenario was mediated by harmony-seeking at the ideal-self level (Hashimoto and Yamagishi, 2013), where high-risk students preferred not to be oriented toward harmony seeking. Similarly, Ishii and Uchida (2016) found that NHR score was correlated with an inclination to deviate from other culturally normative tendencies, such as lower desire to engage in social activities, lower need for belonging, lower levels of interdependence, and being less attentive to context (in spontaneously attending to the vocal tone of spoken words). In sum, students at higher risk of marginalization in Japan demonstrated motivation deviant from the dominant interdependent cultural standards without demonstrating an alternative motivation pattern. However, this effect has not yet been tested among Freeters, who are in reality closer to the edge of socio-economic marginalization. Therefore, in this study we attempt to investigate Freeters' NHR score, compliance motivation and harmony-seeking tendency, and compare that with students' to see if past research's results on high and low-risk students can be extended to the actual Freeter sample.

### Motivation of Freeters

In Merton's (1938) anomie theory, the acceptance and rejection of cultural goals and institutional norms create different types of groups in the social structure. The majority, or *conformists*, are those who accept both cultural goals and institutional means to achieve these goals. In the Japanese context, one important goal is to land a full-time employment, and this notion of success comprises the institutional norms helping to achieve it, such as valuing social harmony, entering an elite university, following the one-time job-hunting process and getting promotion based on seniority. Because the cultural goals and the institutional means are intertwined in the Japanese society (by being successful one needs to have good education, value social harmony, and land a full-time employment), *innovators*, who accepts only the cultural goals but not the institutional means, do not really apply to the Japanese context. Therefore, when the cultural goal is unattainable, people by default become *ritualists*, who continue to follow the institutionalized means without receiving the reward, as is the case of Freeters or part-time job workers. Those who reject both cultural goals and institutionalized means for accomplishing those goals are *retreatists*. *Retreatists* are those who disengage completely from the mainstream society, and in Japan's case NEET and Hikikomori fall into this category (Toivonen et al., 2011).

On the scale of marginalization risk in the Japanese context, Freeters stand between high-risk students and NEET. Following Norasakkunkit and Uchida (2014)'s study that those with higher NHR score scored lower in their compliance motivation to obtain social proof (the culturally dominant norm in Japan), it is thus expected that Freeters in general would have lower WTC in the social proof scenario compared to students. Toivonen et al. (2011) put Freeters in the *ritualists* category, and NEET and Hikikomori in the *retreatists* category in Merton's model. The difference between *ritualists* and *retreatists* is in their acceptance of the institutional norms despite the small possibility of obtaining the rewards. Freeters continue to be engaged in the peripheral labor market, and some still try to land a full-time employment, whereas NEETs and Hikikomori may have given up making this effort to re-integrate into society.

As Toivonen et al. (2011, p. 5) pointed out, in a conformist society like Japan, the flow from mainstream to periphery goes from *conformists* to *ritualists*, and then to *retreatists*. However, whether the flow from *ritualists* to *retreatists* (from Freeter to NEET and Hikikomori) is shaped by the larger social environment or due to personal reasons remain unclear. If the larger social environment shapes this flow of marginalization, then when presented with a grim picture of future job-prospects, where there is little hope of returning to the mainstream society, students might be pushed from *conformists* to *ritualists*, and Freeters from *ritualists* to *retreatists*. In this case, whereas students may continue holding on to the traditional cultural values, Freeters might abandon them and go further down the trail of marginalization.

### Current Study

In the first part of the study, we will compare Freeters' compliance motivation with that of students. Based on past research on compliance motivation of those with high NHR score (Norasakkunkit and Uchida, 2014), our hypothesis is that Freeters would score higher in NHR and lower in WTC social proof and harmony-seeking tendency compared to students. Next, we will examine the effect of the larger socio-economic environment on compliance motivation across both samples first, and then examine the effect of priming among students and Freeters separately. To do this, we will randomly prime both students and Freeters to either a marginalization risk situation (where it is extremely difficult to get a satisfying job due to economic reasons) or a non-marginalization situation (where the job prospects are good for the next decade). We will also explore their compliance motivation for social proof (interdependent cultural value) and self-consistency (independent cultural value) compared to the control group sample. We expect the interaction that students primed with marginalization risk (difficulties finding a full-time job) will be pushed from *conformists* to *ritualists*, thus maintaining high compliance motivation in the social proof scenario and decreasing compliance motivation in the self-consistency scenario, becoming more like typical Japanese, whereas Freeters who are already in the periphery of society might be pushed from *ritualists* to *retreatists*, decreasing their compliance motivation for both social proof and self-consistency.

This study aims to investigate the effect of job-hunting prospects at the macro level on individual motivations at the micro level, more specifically whether it would undermine their compliance motivation for social proof or prompt them to make more effort to “fit in” to mainstream society. The usage of priming helps investigate a mindset under risks of socio-economic marginalization. As most previous studies on NEET/Hikikomori risk and cultural deviation compared high-risk and low-risk people in student samples, the inclusion of Freeter samples in this study examines whether previous results were generalizable beyond students, and sheds light on the motivation tendencies of a growing population on the actual edge of marginalization.

## Materials and Methods

Participants completed an online survey questionnaire in the lab (for student sample) or on the internet (for Freeter sample). They were randomly assigned to either the marginalization risk (poor job prospects) or the non-marginalization condition (good job prospects), and were given an article from the internet to read about future job prospects in Japan. In the student sample, they were asked to spend 10 min (timed by experimenter) reflecting on what they just read and writing down how they would feel if the job market they face when they are job-hunting is as described in the article. In the Freeter sample recruited online, they were asked to write at least five sentences about how they would feel toward the situation. They then continued the survey, starting with the manipulation check questions on how happy (positive affect) and worried (negative affect) they were about the situation (1 = Not at all, 5 = Very much). This was followed by the NEET-Hikikomori Risk Spectrum (NHR), the Willingness to Comply (WTC) scenarios (randomly assigned to either social proof or self-consistency, between-subject DV), the Ideal-Self questionnaire, and demographic questions.

### Participants

#### Student Sample in the Lab

The student sample came from Kyoto University. Sixty-five students from 1st year undergraduate to 3rd year PhD signed up to participate in the experiment. Twenty-one were female, and 53 were undergraduate students. They aged between 18 and 37 (*M* = 21.72, *SD* = 3.13). Thirty-three were randomly assigned to the marginalization risk priming condition, and 32 were assigned to the WTC social proof scenario. They were scheduled to come to the laboratory, and were told that the study will last an hour with 1,500-yen worth of book coupons as compensation, which includes participation in another study conducted. After informed consent was obtained, participants were led to a computer to generate an identification code that is used to keep participants anonymous. All survey questions were in Japanese as used in previous research. Upon completion, we debriefed participants and explained that the internet article about job-hunting was made up for the purpose of the experiment.

#### Freeter Sample

Sample from the Freeter population came from *Lancers*, a large cloud sourcing job service website widely used in Japan. In order to recruit real Freeter sample, we set up a pre-screening session. In the pre-screening survey, 600 participants were recruited to complete a 5 min survey with the NHR and questions (pre-score) about their age, gender and occupation. The survey ended asking participants to create an ID that could be linked to their Lancers ID. Full-time workers, homemakers, those on temporary leave from work and over 45 years-old were excluded, which leaves 152 participants eligible for the main survey. Out of the 152 who were contacted 2 weeks after completion of the pre-survey, we had 110 completed responses. To ensure the priming procedure, we discarded 34 of those who did not follow the instructions and wrote less than five sentences after reading the priming article, as well as 2 who indicated that they were currently students, and the remaining sample for our analyses consists of 74 subjects. They aged between 22 and 44 (*M* = 32.7, *SD* = 6.71), and 50 were female. Forty-three were assigned to the marginalization risk priming condition, and 36 were assigned to the WTC social proof scenario. Participants were rewarded with a payment of 200 Japanese yen for the completion of a 15 min online survey.

#### Control Group Sample

In addition to the main survey participants, we recruited control group students from Kyoto University via the same advertising procedures. Twenty-three students completed the survey online (7 female, age 18–29, *M* = 20.7, *SD* = 2.46), which consists of all measurement scales except for the priming scenario and the affect questions. Seven students completed WTC for the social proof scenario. They were then scheduled to come to the lab to receive 500-yen worth of book coupons. We also recruited control group Freeters online via *Lancers* by recruiting 60 participants for a short survey, and kept the data of 22 who were Freeters (11 female, age 20–45, *M* = 38, *SD* = 6.43). The procedure for control group Freeters was the same as for control group students, except that we added questions measuring affect for Freeters, and 14 of them were in the WTC social proof scenario. The research has obtained IRB approval from Gonzaga University, and we conducted the study in accordance with the ethical guidelines of Kyoto University and the Japanese Association of Psychology.

### Materials

#### Priming Scenarios

Participants were asked to read a scenario that represented either the marginalization risk condition or the non-marginalization condition (between-subject factor). For the marginalization risk condition, the scenario stated that the job-hunting prospects are poor even for those who graduate from elite universities, and that it would get worse in the future. In contrast, the non-marginalization scenario stated that the economic situation is recovering and that the job-hunting prospects will get even better in the future (see Appendix I).

#### Willingness to Comply (WTC)

The questionnaire asks how much one is likely to comply with completing a 40 min marketing survey on a scale of 0 (very low likelihood) to 8 (very high likelihood). Two scenarios are given (social proof and self-consistency; between-subject factor). This was followed by questions asking how likely one is to comply if (1) all classmates have complied (social proof)/in the past one has always complied (self-consistency); (2) half of the classmates have complied (social proof)/in the past one has complied half of the time (self-consistency); (3) none of the classmates have complied (social proof)/in the past one has never complied (self-consistency). The scenarios were identical with the ones used in the previous study (Norasakkunkit and Uchida, 2014). WTC score is calculated by subtracting the score of question 3 from that of question 1 (Cialdini et al., 1999). Higher WTC score in the social proof scenario is considered higher motivation to conform to mainstream Japanese cultural values, and higher WTC score in the self-consistency scenario is considered higher motivation to adapt to individualistic cultural values.

#### NEET-Hikikomori Risk Spectrum (NHR)

We used the scale by Uchida and Norasakkunkit (2015). It consists of 27 items divided into three factors: (1) Freeter lifestyle preference, which is the preference of not having to work hard in full-time employment (e.g., I don't think it is necessary to find a job immediately; 14 questions, α = 0.83); (2) Lack of self-competence, which is low self-esteem in both personal capabilities and relations (e.g., I feel that communicating with others is hopelessly difficult for me; 11 questions, α = 0.83); and (3) Unclear ambitions for the future, which is not having a clear goal (e.g., I don't quite know what I want to do in the future; 2 questions, α = 0.79). Participants rate how much they agree with the items on a 7-point Likert scale from “Completely disagree” to “Completely agree.”

#### Ideal-Self

We used the scale by Hashimoto and Yamagishi (2013). The questionnaire consists of 18 items divided into three factors: Independence (α = 0.80), Harmony-seeking (α = 0.69), and Rejection avoidance (α = 0.76). Each item asks participants to rate how much a statement describes their ideal self on a 7-point scale from “Does not describe at all” to “Describes very well.” Here we mainly focus on the score in harmony-seeking, as it has been shown to mediate NHR score (Norasakkunkit and Uchida, 2014).

## Results

### Preliminary Analysis

The preliminary analysis was to do a manipulation check on whether participants' emotions were affected after reading the article. The student control group, which was collected earlier, did not include the manipulation check, but we included those questions for the Freeter control group collected later. The comparisons with control groups is included in the following sections. We subtracted negative affect (how worried you are about the situation) from positive affect (how happy you are about the situation) and expected that those in the marginalization risk priming condition would score lower on affect than those in the non-marginalization condition, and that this effect would be stronger among students who still face the challenge of job-hunting than among Freeters. Because there was a significant age and gender ratio difference between the two sample groups, we treated age and gender as co-variates in all analyses. A 2-way (2 priming conditions × 2 samples) ANCOVA was run to compare participants' affect about the situation, controlling for age and gender. A significant main effect of priming conditions was found, showing that those in the marginalization risk condition (*n* = 76, *M* = −2.46, *SE* = 0.19) scored lower on affect than those in the non-marginalization condition [*n* = 63, *M* = −0.41, *SE* = 0.21; *F*_(1, 133)_ = 51.37, *p* < 0.001, partial η^2^ = 0.279]. There was also an interaction effect [*F*_(1, 133)_ = 14.17, *p* < 0.001, partial η^2^ = 0.096], and *post-hoc* LSD tests showed that students in the non-marginalization condition (*n* = 32, *M* = 0.35, *SE* = 0.34) tended to score higher than Freeters in the same condition (*n* = 31, *M* = −1.17, *SE* = 0.37, *p* = 0.009). No difference was observed in the marginalization risk condition (students *n* = 33, *M* = −2.79, *SE* = 0.33; Freeters *n* = 43, *M* = −2.13, *SE* = 0.27; *p* = 0.162). This may be reasonable, given that a bright picture of future job prospects may benefit the students more than it would the Freeters who are already in the periphery. In sum, the marginalization risk priming negatively affected both Freeters and students, and the non-marginalization priming positively affected the students more than the Freeters.

### NHR and Harmony-Seeking at the Ideal-Self Level: Comparison of Student and Freeter Samples

Our first hypothesis was a comparison of NHR scores and harmony-seeking tendencies between students and Freeters (*N* = 184) before moving on to examine their compliance motivation. Given that Freeters stand at a closer end to marginalization, we expected Freeters to score higher in NHR than students. Following Norasakkunkit and Uchida (2014)'s findings that high NHR risk students scored lower in the WTC social proof scenario (henceforth called WTC social proof) and harmony-seeking at the ideal-self level, we expected Freeters to follow this tendency. A 2-way ANCOVA (2 samples × 3 priming conditions) was conducted to determine a statistically significant difference between Freeters and students on NHR, controlling for age and gender. A significant main effect of sample showed that Freeters (*n* = 96, *M* = 111.19, *SE* = 3.03) scored higher than students [*n* = 88, *M* = 91.19, *SE* = 3.06; *F*_(1, 176)_ = 14.63, *p* = 0.001, partial η^2^ = 0.077], confirming that Freeters are on the further end of marginalization. A posteriori power analysis showed that this result has enough power for a medium effect size (*f* = 0.25 resulted in 1-β = 0.92), but not enough power for a small effect size (*f* = 0.10 resulted in 1-β = 0.27). There was also a main effect of priming conditions [*F*_(2,176)_ = 6.66, *p* = 0.002, partial η^2^ = 0.070], but *post-hoc* LSD tests showed that it was the control group (*n* = 45, *M* = 109.74, *SE* = 3.11) who scored higher on NHR than both the marginalized (*n* = 76, *M* = 98.50, *SE* = 2.41; *p* = 0.005) and the non-marginalized group (*n* = 63, *M* = 95.31, *SE* = 2.61; *p* < 0.001). No interaction was found [*F*_(2,176)_ = 0.66, *p* = 0.518, partial η^2^ = 0.007].

For harmony-seeking at the ideal-self level, a 2-way ANCOVA (2 samples × 3 priming conditions) was conducted, controlling for age and gender. In contrary to our hypothesis, no main effect of sample was found [student *n* = 88, *M* = 32.64, *SE* = 0.96; Freeter *n* = 96, *M* = 31.33, *SE* = 0.95; *F*_(1, 176)_ = 0.63, *p* = 0.428, partial η^2^ = 0.004]. However, there was a main effect of priming [*F*_(2,176)_ = 3.88, *p* = 0.022, partial η^2^ = 0.042], and *post-hoc* LSD tests showed again that it was the control group (*n* = 45, *M* = 30.07, *SE* = 0.97) who scored lower on harmony-seeking than the non-marginalization group (*n* = 76, *M* = 33.63, *SE* = 0.82; *p* = 0.006). In sum, our sample confirmed the first hypothesis that Freeters who are on the further end of socio-economic marginalization score higher on NHR. However, in contrast to what previous study suggested, they did not seem to deviate from the mainstream cultural values in harmony-seeking at the ideal-self level. In addition, our control group sample scored higher on NHR and actually exhibited high NHR behaviors consistent with past research, scoring lower on harmony-seeking tendencies^1^.

### Effect of Marginalization Risk Priming on Compliance Motivation

**Figure 1:**
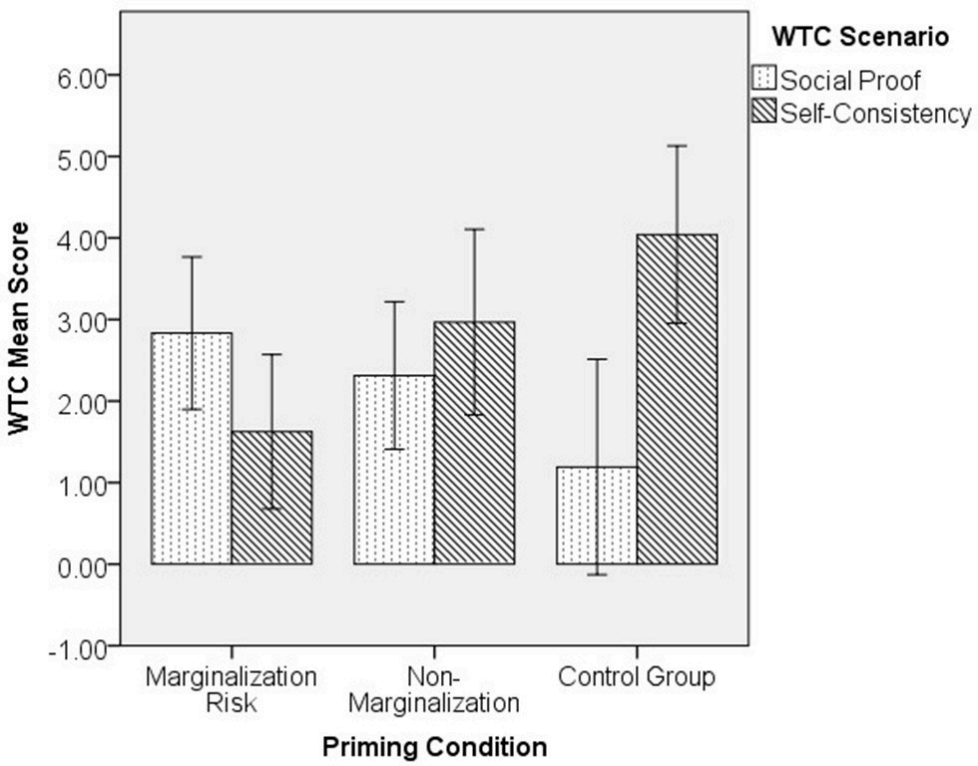
Priming conditions and compliance motivation across samples.

To examine the effect of priming on students' and Freeters' compliance motivation, we conducted a 3-way ANCOVA (3 priming groups × 2 samples × 2 WTC scenarios, controlling for age and gender). Results showed a main effect of sample, where students (*n* = 88, *M* = 3.48, *SE* = 0.41) scored higher on compliance in general than Freeters [*n* = 96, *M* = 1.53, *SE* = 0.40; *F*_(1, 170)_ = 7.86, *p* = 0.006, partial η^2^ = 0.044]. A posterior power analysis showed that our results were powerful enough for a medium effect size (*f* = 0.25 resulted in 1-β = 0.92), but not enough for a small effect size (*f* = 0.10 resulted in 1-β = 0.27). A significant 2-way interaction was also found between priming conditions and WTC scenarios [*F*_(2,170)_ = 5.72, *p* = 0.004, partial η^2^ = 0.063]. A posterior power analysis showed that this result was powerful enough for a medium effect size (*f* = 0.25 resulted in 1-β = 0.86), but not enough for a small effect size (*f* = 0.10 resulted in 1 – β = 0.21). *Post-hoc* LSD tests showed that, in WTC self-consistency, those primed with marginalization risk (*n* = 40, *M* = 1.60, *SE* = 0.44) scored lower than both those primed with non-marginalization (*n* = 31, *M* = 3.02, *SE* = 0.49, *p* = 0.032) and the control group (*n* = 24, *M* = 3.62, *SE* = 0.59, *p* = 0.006). In WTC social proof, the marginalization risk group (*n* = 36, *M* = 2.95, *SE* = 0.46) scored slightly higher than the control group (*n* = 21, *M* = 1.52, *SE* = 0.63; *p* = 0.071), but the difference was not significant. Within each priming group, *post-hoc* LSD tests showed that the marginalization risk group participants scored higher in WTC social proof (*n* = 36, *M* = 2.95, *SE* = 0.46) than in WTC self-consistency (*n* = 40, *M* = 1.60, *SE* = 0.44, *p* = 0.033). The control group participants showed the opposite trend, scoring higher in WTC self-consistency (*n* = 24, *M* = 3.62, *SE* = 0.59) than WTC social proof (*n* = 21, *M* = 1.52, *SE* = 0.63, *p* = 0.015). No difference was observed in the non-marginalization group (*n* = 32, *M* = 2.33, *SE* = 0.48 for social proof and *n* = 31, *M* = 3.02, *SE* = 0.49 for self-consistency; *p* = 0.315). In sum, contrary to our hypothesis, marginalization risk increased compliance motivation for social proof and decreased compliance motivation for self-consistency across samples (see Figure 1). However, at a closer look it was the control group's motivation pattern that deviated from the expected cultural norms (i.e., scoring higher in independence-oriented motivation rather than interdependence-oriented motivation), and marginalization risk priming reversed that pattern. No significant 3-way interaction was observed^2^.

### Effect of Marginalization Risk Priming on Students' Compliance Motivation

**Figure 2:**
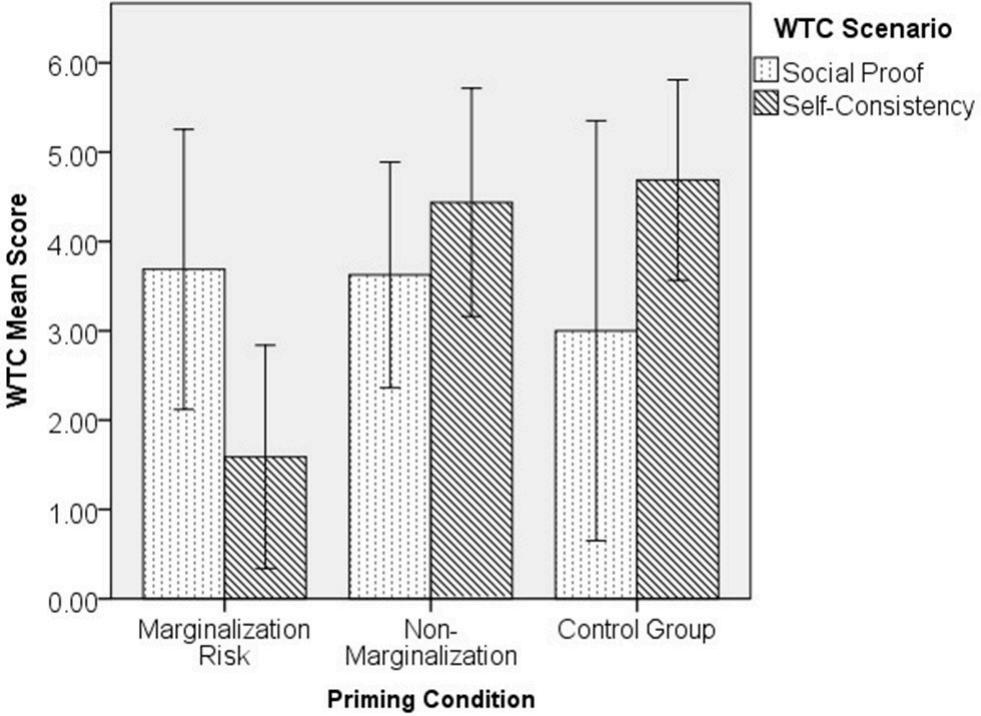
Priming conditions and compliance motivation among students.

To further explore the effect of marginalization risk on each sample, we conducted a 2-way ANCOVA (3 priming groups × 2 WTC scenarios) on students' WTC, controlling for age and gender (*n* = 88). Results showed a significant interaction between priming and WTC scenarios [*F*_(2,80)_ = 4.68, *p* = 0.012, partial η^2^ = 0.105]. A posterior power analysis showed that our results were not powerful enough for a medium effect size (*f* = 0.25 resulted in 1-β = 0.53). *Post-hoc* LSD tests showed that the difference was in WTC self-consistency, where students primed with marginalization risk (*n* = 17, *M* = 1.48, *SE* = 0.66) scored lower than both students primed with non-marginalization (*n* = 16, *M* = 4.49, *SE* = 0.66, *p* = 0.002, Cohen's *d* = 1.11) and control group students (*n* = 16, *M* = 4.85, *SE* = 0.67, *p* = 0.001, Cohen's *d* = 1.28). No between-group differences were found for WTC social proof. Within each priming group, only the students in marginalization risk priming group showed a significant difference between each WTC scenarios, where they scored lower in WTC self-consistency (*n* = 17, *M* = 1.48, *SE* = 0.66) relative to WTC social proof (*n* = 16, *M* = 3.72, *SE* = 0.66, *p* = 0.019, η^2^ = 0.066). This showed that the risk of marginalization did not deviate students' compliance motivation from mainstream society, but instead decreased their independence-oriented motivation (see Figure 2).

### Effect of Marginalization Risk Priming on Freeters' Compliance Motivation

**Figure 3:**
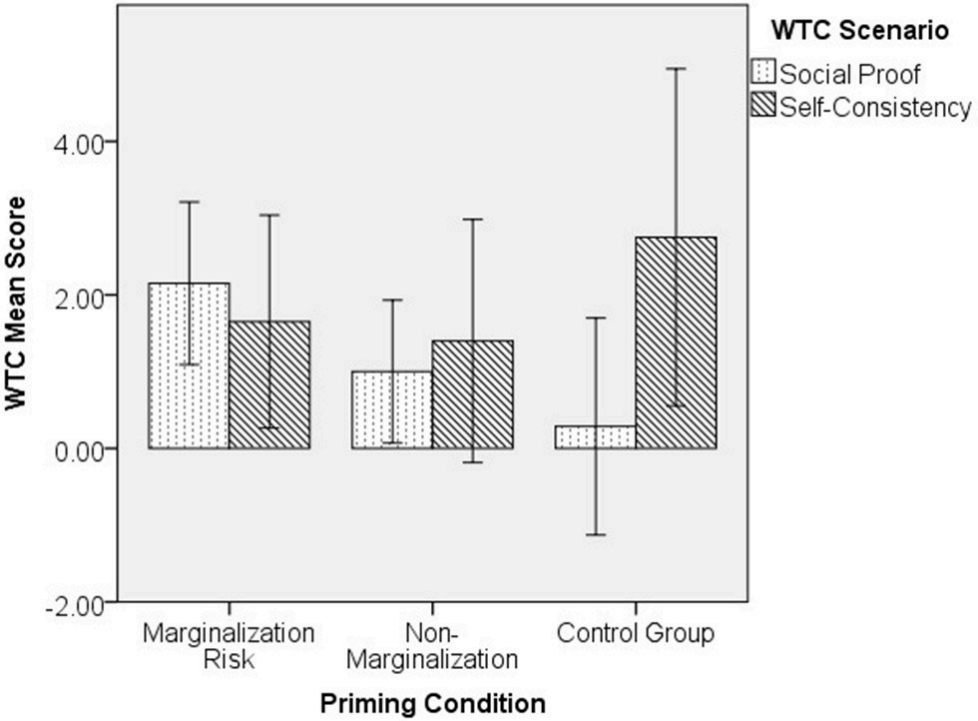
Priming conditions and compliance motivation among Freeters.

To explore the effect of marginalization risk priming on Freeters we conducted a one-way ANCOVA on affect and a 2-way ANCOVA (3 priming groups × 2 WTC scenarios) on compliance motivation, controlling for age and gender (*n* = 96)^3^. For WTC scores, no significant results were found [*F*_(2,88)_ = 1.90, *p* = 0.156, partial η^2^ = 0.041 for interaction], but because of the relatively small sample size in each group and the close to medium effect size, we examined each compliance motivation separately with one-way ANCOVA. Results showed that for WTC social proof (*n* = 50), there was a significant difference across the 3 groups [*F*_(2, 45)_ = 3.26, *p* = 0.048, partial η^2^ = 0.126]. *Post-hoc* LSD tests showed that those in marginalization risk condition (*n* = 20, *M* = 2.28, *SE* = 0.54) scored higher than the control group (*n* = 14, *M* = 0.04, *SE* = 0.66; *p* = 0.016, Cohen's *d* = 0.75). No difference was found for WTC self-consistency [*F*_(2, 41)_ = 0.46, *p* = 0.635, partial η^2^ = 0.022]. This shows that marginalization risk priming increased rather than decreased Freeters' compliance motivation for social proof compared to the control group (see Figure 3). However, a posterior power analysis for the one-way ANCOVA with *f* = 0.25 resulted in 1-β = 0.31, which was not sufficient to demonstrate a medium effect size given our small sample.

## Discussion

The first part of our study tried to follow through past research's claims that NHR is a measurement of marginalization risk, and that those at the further end of marginalization have less motivation to conform to traditional cultural norms (Norasakkunkit and Uchida, 2011, 2014; Ishii and Uchida, 2016). We did not conduct a priori power analyses to determine the sample size needed, but based on past research on NHR tendencies among working and non-working populations (Uchida and Norasakkunkit, 2015), we estimated a medium effect size, and posterior power analyses showed that with our full sample size our power was sufficient to demonstrate the expected medium-size effect. Freeters, who are on further end of socio-economic marginalization, scored higher in NHR and lower in WTC compared to students. However, this effect was not mediated by harmony-seeking at the ideal-self level (Hashimoto and Yamagishi, 2013), where there was no difference between students and Freeters. This means that, in contrast to high-risk students in previous studies, the decreased motivation among Freeters to comply toward in-group members is not due to their disbelief in the mainstream cultural values of harmony-seeking. As *ritualists* in Merton's model, they may still value and want to follow the mainstream cultural norms (harmony-seeking), but the reality of socio-economic marginalization may demotivate them to do so (lower WTC social proof), since they are not reaping the rewards. From a goal-regulation viewpoint, people tend to withdraw from pursuing goals that are not going well (Carver and Scheier, 1998). There is the possibility that the failure to enter mainstream society may have reduced their general compliance motivation (whether in the service of maintaining perceived self-consistency or in the service of conformity with in-group members) due to learned helplessness (Seligman, 1975), keeping them from having both independent-oriented and interdependent-oriented motivation patterns. One thing to be noted is that in our study most participants were primed to think about good or bad job-hunting prospects, thus their subsequent reactions in NHR score and WTC scenarios may be affected by the process and cannot be directly compared with those obtained in previous studies. More studies with Freeter samples in comparison with student samples will help confirm the relationship between actual marginalization, NEET-Hikikomori risk, and compliance motivation in social proof among Japanese young adults.

Our second question exploring the effect of marginalization risk priming showed that, in contrary to our assumption, when primed to think about poor job-hunting prospects, both students and Freeters have higher motivation to comply for social proof and lower motivation to comply for self-consistency. More specifically, students dropped their motivation to maintain self-consistency, and Freeters increased their motivation to obtain social proof compared to the control group samples. Our exploratory analyses of the student sample showed that students primed with non-marginalization and control group students scored higher in self-consistency and lower in social proof, and it is the students primed with marginalization risk who behaved like low-risk students in the previous study (Norasakkunkit and Uchida, 2014). One possible explanation is the difference in affect. Ashton-James et al. (2009) have demonstrated that when in a positive affect people tend to be more willing to go against cultural norms and try new things (i.e., being more interdependent in America and more independent in Japan), whereas when in a negative affect they tend to hold on to the familiar cultural norms. It is possible that students primed with non-marginalization felt happier, thus were more willing to have a different motivation pattern (i.e., self-consistency). Another explanation is that, as we hypothesized, our student sample in the marginalization risk priming condition fall under the *ritualists* category, who “conform to legitimate means but have little hope for culturally expected rewards” (Toivonen et al., 2011, p. 4). Therefore, when reminded of a highly competitive but less rewarding situation in the future, they still continued their effort to fit into the mainstream society by complying with their peers, but were less motivated to maintain perceived self-consistency, as the same efforts in the past may seem to lose their meaning in the current situation. Since the non-marginalization priming condition conveys the message that they are doing well in the current situation, this may motivate them to continue behaving as they did in the past (thus scoring higher in WTC self-consistency). On the other hand, the marginalization risk priming condition conveys the message that things are not going as well as they planned, so they might feel the need to change the strategy in order to cope with the current situation, thus scoring lower in WTC self-consistency. Further studies on the relationship between socio-economic marginalization risk and its effect on self-consistency and behavior changes will help clarify how the threat is perceived among students.

We also explored the effect of marginalization risk priming on Freeters. The results showed that Freeters primed with marginalization risk scored higher instead of lower in WTC social proof compared to those in the other conditions. Freeters in the non-marginalization condition scored somewhere between the two groups. Again, this tendency may be explained by affect (Ashton-James et al., 2009), as Freeters in the marginalization risk group were indeed feeling more negative, which may have prompted them to hold on to the mainstream cultural norms. It is also possible that Freeters have interpreted non-marginalization priming in a different way than did students. As opposed to students who have not yet entered the mainstream society, they may not feel the benefit from the improvement of the economic situation at the macro level given their status in the periphery. Thus, for Freeters, whereas marginalization risk priming may have reminded them of their own situations, non-marginalization priming may not really relate to them since they cannot reap the benefits, so their affect did not change and they remain demotivated to comply in both scenarios. Another explanation is that culturally deviant motivation or lack of motivation due to both the unattainability of goal pursuit (Carver and Scheier, 1998) and their marginalized status in society is most pronounced when Freeters are insulated from the reality of their socio-economically marginalized status. Nevertheless, when reminded that their survival is at stake, instead of giving up the institutionalized means and withdrawing, they actually strive harder to become more mainstream-oriented. This shows that perhaps short-term marginalization risk still pushes young people in Japan toward *ritualists* behavior, and it is long-term marginalization that eventually leads them to become *retreatists*. At the group level, for Freeters marginalization risk priming seems to have served as a “warning sign,” and once being reminded of their status they made an effort to “fit in” to mainstream society and not to become *marginalized* (Berry and Sam, 1997). Thus, being placed at marginalization risk is perhaps not equivalent to being marginalized in reality, and future studies are needed to provide insight to the reality of demotivation in a real-life marginalized situation to examine the factors that prompt individuals to move from *ritualists* to *retreatists*.

There are several limitations in our study. First, in terms of the experimental procedure, our student sample came to the laboratory to be primed and to complete the survey online, whereas our Freeter sample's data was collected solely via the internet. Although both samples wrote about their thoughts regarding the priming article, we cannot rule out the possibility that the different effect of priming observed between Freeters and students may be due to the priming procedures, which is more thoroughly executed for students than for Freeters. Second, participants from different samples, as explained above, may interpret marginalization risk priming differently. While for students it may be a confirmation that they are doing well and should continue their efforts, for Freeters it may seem ironic or unbelievable to them that the macro situation is getting better yet they are still in the peripheral labor market. Third, a main difference between our samples was age, which may affect people's compliance motivation. In Japan where one gains more respect as one raises in seniority, it is possible that those who are older simply feel less obliged to comply. Fourth, our student sample is solely from Kyoto University, one of the most elite in Japan, thus it is possible that the marginalization risk priming does not really consists of a threat, so there are limitations to the generalizability of the results. Future studies need to collect data from differently ranked universities to see how the macro job-hunting prospects may affect the motivation patterns of students from different parts of the social hierarchy. Fifth, our control group sample for both students and Freeters was collected separately from the experiment sample, which may induce a selection bias. As a consequence, the control group samples showed tendencies of marginalization, scoring higher in NHR and lower in WTC and harmony-seeking at the ideal-self level, which may or may not be representative of the student population of Kyoto University. Sixth, both priming and willingness to comply were assessed via participants' self-report responses to survey questions, and for manipulation check we only measured affect, but marginalization risk priming may not have primed feelings of marginalization. In the future, simulations of ostracism in the laboratory setting (using isolation games, for example) or data collection after a real job interview with a confederate demanding for compliance to fill a survey may provide insights to how a real-life marginalizing (or non-marginalizing) situation may affect people's compliance motivation and attitude. Seventh, this study does not include the real NEET and Hikikomori sample, thus we cannot assess the effect of long-term vs. short-term alienation. It is possible that Freeters remain *ritualists* in the beginning, but after decades of marginalization gradually became *retreatists*. It is also possible that individual factors are more involved in determining whether a marginalizing situation at the macro level pushes one to become *retreatists*. Future studies with real NEET and Hikikomori sample are needed to understand the transition from *ritualists* to *retreatists*. Cross-cultural comparisons with data collected in an independent culture may also help clarify the effect of socio-economic marginalization and culturally deviant compliance motivation patterns.

In conclusion, as Berry and Sam proposed in the acculturation model, adaptations take place when entering a new cultural context, and depending on various factors, “cultural fit” can be achieved via *assimilation* or *integration* orientation from the person and an accepting attitude from the society. Other times, however, “cultural fit” is not achieved with *separation*/*segregation* and *marginalization*, resulting in intergroup conflicts and psychological stress. The increasing number of Freeter, NEET and Hikikomori in the twentieth century Japan can be interpreted as a growing abundance of those lacking “cultural fit” in the society, which results from the high rigidity of formal education and employment structure in Japan, leaving few alternatives and weak governmental support for those who fail. This research attempted to see the effect of marginalizing situations in the socio-economic setting and evaluate its effect on both students' and Freeters' motivation and attitudes. It showed that, as Toivonen et al. (2011) predicted, when the *conformist* path seems unavailable, Japanese young people by default become *ritualists* (Merton, 1938). Students in the marginalization risk priming condition were not less motivated to adhere to cultural norms, but were less motivated to be self-consistent. Freeters in the same condition were actually more motivated to comply with in-group members. In an interdependent culture, such as Japan where one's personal identity is constructed by its belonging to a group, the lack of good job prospects in the future can mean risks of ending up with a marginalized identity. Yet even when this threat is present (or being reminded of its presence), neither students nor Freeters lowered their motivation to adhere to cultural norms. In other words, they were all striving for cultural fit despite the situation of marginalization risk. While urging for the rigid societal structure to adapt to globalizing pressure in order to offer more chances to the younger generation (students), social and psychological support is also needed for those already in the periphery (Freeters). This includes support for the job-hunting process for those who wish to enter the core labor market (*assimilation*), and support for constructing a positive identity for those who choose not to enter (*integration*). A more accepting attitude from the mainstream society will satisfy the need of belonging and eliminate the prejudice of Freeters, give them a chance to fit in, and decrease the risk of any individuals to go further down the spectrum of marginalization.

## Author Contributions

I-TH-CL conducted the research, which was designed together with YU and VN. All authors participated in the data analyses. I-TH-CL drafted the manuscript, and YU and VN helped with editing and commenting.

### Conflict of Interest Statement

The authors declare that the research was conducted in the absence of any commercial or financial relationships that could be construed as a potential conflict of interest.

## Footnotes

^1^As the control group sample was collected online separately from the priming studies, we did analyses with only the control group samples to examine the baseline difference between student and Freeter samples. We conducted one-way ANCOVAs to examine the difference in their NHR score and their harmony-seeking at the ideal-self level, and a 2-way ANCOVA to examine their difference in WTC scores across both scenarios, controlling for age and gender. Results showed a difference in NHR score [*F*_(1, 41)_ = 4.11, *p* = 0.049, partial η^2^ = 0.091], with Freeters (*n* = 22, *M* = 124.63, *SE* = 8.59) scoring higher than students (*n* = 23, *M* = 93.62, *SE* = 8.28), but no difference was found in harmony-seeking [*F*_(1, 41)_ = 0.11, *p* = 0.742, partial η^2^ = 0.003]. The 2-way ANCOVA showed a main effect of WTC scenario, with both samples scoring higher on self-consistency (*n* = 24, *M* = 3.66, *SE* = 0.56) than social proof [*n* = 21, *M* = 1.63, *SE* = 0.60; *F*_(1, 39)_ = 6.17, *p* = 0.017, partial η^2^ = 0.137]. These and results from our full-model analyses on NHR and harmony-seeking shows that our control group sample may have higher NHR risk than those in other conditions, probably due to selection bias.

^2^We conducted a multiple linear regression to explore how NHR score instead of sample (Freeters vs. Students) may predict each compliance scenario. Because of the self-selection bias in the control group sample who scored higher in NHR, we excluded them from the regression (*n* = 139). The WTC score was regressed on: (1) Gender (1 = male, 0 = female); (2) Age; (3) NHR score; (4) Priming condition (1 = marginalization risk, 0 = non-marginalization). The result was non-significant [*F*_(5, 133)_ = 1.94, *p* = 0.093] with an *R*^2^ = 0.07. This shows that NHR scores may differ between the samples, but NHR score alone cannot replace the sample variable and predict compliance motivation, thus for our model we included all three priming groups and both samples as independent variables for comparison.

^3^For affect, there was a significant effect of priming [*F*_(2, 91)_ = 6.23, *p* = 0.003, partial η^2^ = 0.121]. *Post-hoc* LSD tests showed that those in the marginalization risk condition (*n* = 43, *M* = −1.97, *SE* = 0.27) scored lower than both the non-marginalization group (*n* = 31, *M* = −1.05, *SE* = 0.31; *p* = 0.028, Cohen's *d* = 0.25) and the control group (*n* = 22, *M* = −0.35, *SE* = 0.38; *p* = 0.001, Cohen's *d* = 0.30).
