# Supplementary material for: Socio-Economic Marginalization and Compliance Motivation Among Students and Freeters in Japan
Source: Front Psychol. 2019 Feb 26;10:312. doi: 10.3389/fpsyg.2019.00312 (PMC6399110; doi:10.3389/fpsyg.2019.00312)
Supplement: Supplementary file 1 [file Data_Sheet_1.pdf]

Appendix I

Marginalization Risk Condition

Crowds of young people, somewhat ill-at-ease in their matching black suits, are a common sight at this time of year in the business districts of Tokyo. The height of the spring recruitment season is upon us again, and another batch of young hopefuls is on the hunt for job offers that will see them enter the workforce in April 2016. People may say that the job market seems fine, but economist professionals projected that within a few years a fair number of students will fail to land an offer of full-time employment. In addition, 50% of students cannot get their preferred job or are not satisfied with the job they have. With huge numbers of job seekers applying to large firms, even for the students in top elite university, the chances of getting a stable job are becoming increasingly pessimistic compared to job prospects in years past. The challenge young people face in the job market is expected to become progressively worse every year for at least another decade.

どこか不安げな様子の、同じような黒いスーツに身を包んだ若者の集団は、この時期の東京のビジネス街ではよく見られる光景だ。春の採用活動はピークを迎え、今年もまた前途有望なたくさんの若者たちが 2017 年の 4 月に入社するための就職活動の時期に入る。求人市場の状況は良好だと思われているようだが、経済の専門家は、この 2～3 年のうちはかなりの数の学生が正規雇用での採用にたどり着くことができないと予想している。加えて約 50％の学生が希望の仕事に就くことができない、もしくは就いた仕事に満足できていない。大企業に就職希望者が殺到し、たとえトップエリート大学の学生であっても、安定した職に就くことができる可能性は、過去数年と比べてますます悲観的になっている。若者が求人市場で直面する困難な状況は、少なくとも今後 10 年の間、年々悪化の一途をたどることが予想されている。

Non-Marginalization Condition

Crowds of young people, in their matching black suits, are a common sight at this time of year in the business districts of Tokyo. The height of the spring recruitment season is upon us again, and another batch of young hopefuls is on the hunt for job offers that will see them enter the workforce in April 2016. The Japanese economy got back on the recovery track since the recession in 2008. According to the latest survey, the consensus among most economists is that job prospects for young college graduates have significantly improved in the last few years and is projected to be increasingly optimistic for at least another decade. In fact, economists have concluded that 2015 will be "a year of economic upturn" for Japan. This is good news for current college students who are facing record number of openings for stable jobs in large companies compared to that of previous years.

同じような黒いスーツに身を包んだ若者の集団は、この時期の東京のビジネス街ではよく見られる光景だ。春の採用活動はピークを迎え、今年もまた前途有望なたくさんの若者たちが 2017 年の 4 月に入社するための就職活動の時期に入る。日本の経済は 2008 年の景気後退以来、回復基調に戻っている。最新の調査によると、ほとんどの経済学者たちの共通の見解は大卒の若者の就職見通しがここ数年で大幅に改善しており、少なくともこの十年は楽観視できるとのことだ。実際、経済学者は 2016 年は日本の「景気回復の年」になると結論付けている。これは過去数年に比べて記録的に多い、大企業の安定した雇用の機会に直面している大学生にとってうれしい知らせである。
